# The contribution of* YTHDF2* gene rs3738067 A>G to the Wilms tumor susceptibility

**DOI:** 10.7150/jca.62154

**Published:** 2021-08-26

**Authors:** Zhiyuan Wang, Zhenjian Zhuo, Linyan Li, Rui-Xi Hua, Li Li, Jiao Zhang, Jiwen Cheng, Haixia Zhou, Suhong Li, Jing He, Shan Yan

**Affiliations:** 1 Department of Pathology, The First Affiliated Hospital of Kunming Medical University, Kunming 650031, Yunnan, China.; 2 Department of Pediatric Surgery, Guangzhou Institute of Pediatrics, Guangdong Provincial Key Laboratory of Research in Structural Birth Defect Disease, Guangzhou Women and Children's Medical Center, Guangzhou Medical University, Guangzhou 510623, Guangdong, China.; 3 Department of Clinical Laboratory, Yunnan Key Laboratory of Laboratory Medicine, the First Affiliated Hospital of Kunming Medical University, Kunming 650032, Yunnan, China.; 4 Kunming Key Laboratory of Children Infection and Immunity, Yunnan Key Laboratory of Children's Major Disease Research, Yunnan Institute of Pediatrics Research, Yunnan Medical Center for Pediatric Diseases, Kunming Children's Hospital, Kunming 650228, Yunnan, China.; 5 Department of Pediatric Surgery, the First Affiliated Hospital of Zhengzhou University, Zhengzhou 450052, Henan, China.; 6 Department of Pediatric Surgery, the Second Affiliated Hospital of Xi'an Jiaotong University, Xi'an 710004, Shaanxi, China.; 7 Department of Hematology, The Second Affiliated Hospital and Yuying Children's Hospital of Wenzhou Medical University, Wenzhou 325027, Zhejiang, China.; 8 Department of Pathology, Children Hospital and Women Health Center of Shanxi, Taiyuan 030013, Shannxi, China.; 9 Yunnan Key Laboratory of Stem Cell and Regenerative Medicine, Biomedical Engineering Research Center, Kunming Medical University, Kunming 650500, Yunnan, China.

**Keywords:** Wilms tumor, susceptibility, m^6^A, *YTHDF2*, polymorphism

## Abstract

YTHDF2 is responsible for maintaining the dynamic N^6^-methyladenosine (m^6^A) modification balance and influences a variety of cancers. We tested whether *YTHDF2* gene rs3738067 A>G polymorphism is related to Wilms tumor by genotyping samples of Chinese children (450 cases and 1317 controls). However, the rs3738067 A>G polymorphism showed no statistical significance with Wilms tumor susceptibility. Stratification analysis also revealed that there was no remarkable association of rs3738067 variant AG/GG genotype with Wilms tumor risk in every subgroup (age, gender, and clinical stages). In all, the results indicated *YTHDF2* gene rs3738067 A>G polymorphism could not alter Wilms tumor risk significantly.

## Introduction

Wilms tumor (nephroblastoma) is a typical common seen embryonal kidney cancer in childhood 1. Its prevalence was about 1 in 10,000 children in Western populations 2, whereas 3.3 in one million children in China 3. Wilms tumor usually represents epithelial, undifferentiated/blastemal, and stromal components in varying proportions 4, 5. Moreover, Wilms tumor also displays heterologous elements such as cartilage, osteoid, and neural elements 6-8. This heterogeneity suggests a complexity to the underlying causes of Wilms tumor 9.

Knowledge of the genetic underpinnings of Wilms tumor is growing. In 1990, the *WT1* gene was first cloned as a Wilms tumor suppressor gene 10. Subsequently, mutations in *WTX* and *CTNNB1*, loss of imprinting (LOI), or loss of heterozygosity (LOH) at 11p15 were found to contribute to Wilms tumor development 11-14. Moreover, multiple genetic variants have been identified as Wilms tumor risk loci in genetic association studies 15-18. However, all the identified gene mutations or single nucleotide polymorphisms (SNPs) could only explain a small set of the etiology of Wilms tumor 19, 20. Thus, identification and characterization of more variants are indispensable in better unraveling the full genetic spectrum of Wilms tumor.

N^6^-methyladenosine (m^6^A) is a prevalent internal modification of mRNAs, taking up >80% of all RNA base methylation 21. It regulates the processing, localization, translation, and eventual decay of RNA 22. m^6^A modification is deposited by the methyltransferase complex (writer) composed of METTL3, METTL14, and WTAP 23. Such modification could be reversed by m^6^A demethylases (erasers) including FTO and ALKBH5. Meanwhile, m^6^A associated RNA binding proteins (readers), including YTHDF1-3 and YTHDC1, also function in m^6^A modification by modulating mRNA fate 24, 25. m^6^A modification is highly involved in the carcinogenesis and progression of multiple cancers 26-33. YTHDF2, an m^6^A reader, usually recognizes m^6^A in the 3'-UTR of mRNA, leading to mRNA degradation 34. The contribution of *YTHDF2* gene to oncogenesis has been partly clarified, whereas correlations between *YTHDF2* gene SNPs and Wilms tumor risk have not been analyzed. The current study addresses the association between *YTHDF2* gene SNPs and Wilms tumor risk among children of Chinese ancestry.

## Materials and Methods

### Study subjects

We successfully enrolled 450 cases and 1317 controls to participate in this project. The cases were newly diagnosed and histopathologically confirmed to be Wilms tumor. Control subjects were healthy volunteers with no underlying medical disorder. Controls and cases were frequency-matched by geographically ethnicity, age, and gender. All subjects were Han Chinese children to lessen the genetic background differences. All subjects' guardians provided written consent before accepting any study-related activity. Our research was approved by the Institutional Review Board of the participating hospitals and conducted in accordance with the principles of the Declaration of Helsinki.

### Polymorphism selection and genotyping

The selection of *YTHDF2* gene rs3738067 A>G was based on previously described criteria 35-37. Selection criteria were briefly depicted below: (1) the minor allele frequency (MAF) reported in HapMap was > 5% for Chinese Han subjects; (2) putative functional potentials SNPs located in the 5'- flanking region, exon, 5'- untranslated region (5' UTR), and 3' UTR, which might affect transcription activity or binding capacity of the microRNA binding site; (3) SNPs in low linkage disequilibrium with each other (R^2^<0.8). *YTHDF2* gene rs3738067 A>G is located in transcription factor binding site (TFBS). DNA was extracted from blood using QIAamp DNA Blood mini kit (QIAGEN Inc., Valencia, CA). Genotyping was carried out using TaqMan technology (Applied Biosystems, Foster City, CA) 36. The conditions of reactions were set as follow: pre-read stage at 60 °C for 30 seconds, holding stage at 95 °C 10 minutes, repeated 45 cycles each of denaturation at 95 °C for 15 seconds, annealing and extension at 60 °C for 1 minute. For quality control purposes, 10% of the samples genotyped were randomly duplicated blindly. Quality control analysis showed a concordance rate of 100%.

### Statistical analysis

Chi-square test (for categorical variables) and Student *t*-test (for continuous variables) were employed to evaluate clinical variables differences in the case and control groups. A goodness-of-fit χ^2^ test served to know whether SNP rs3738067 A>G in the controls were agreed with Hardy-Weinberg equilibrium (HWE). The association between rs3738067 A>G and Wilms tumor risk was estimated by odds ratios (ORs) with 95% confidence intervals (CIs) calculated by logistic regression analyses. Statistical analyses were performed with SAS v10.0 (SAS Institute Inc., Cary, NC) and statistical significance was considered when *P* < 0.05.

## Results

### Population characteristics

Baseline characteristics of Wilms tumor cases and controls are shown in **Table S1**. 450 cases and 1317 controls were well matched in terms of age (*P*=0.668) and gender (*P*=0.157). Among the cases, 137 cases (30.44%) were classified into clinical stage I, 122 (27.11%) into clinical stage II, 119 (26.44%) into clinical stage III, 54 (12.00%) into clinical stage IV, and 18 (4.00%) could not be classified.

### Association between *YTHDF2* rs3738067 A>G polymorphism and Wilms tumor risk

A total of 441 cases and 1316 controls were successfully genotyped. The genotype of rs3738067 A>G and its association with Wilms tumor risk is shown in** Table 1**. The *P* value of HWE in control population of rs3738067 A>G was 0.860, meaning no violation of HWE. No significant difference in frequencies of genotype at rs3738067 A>G was observed between cases and controls (AG vs. AA: adjusted OR=0.91, 95% CI=0.73-1.15, *P*=0.444; GG vs. AA: adjusted OR=1.00, 95% CI=0.64-1.54, *P*=0.981; additive: adjusted OR=0.96, 95% CI=0.80-1.14, *P*=0.619; dominant: adjusted OR=0.93, 95% CI=0.75-1.15, *P*=0.491; recessive: adjusted OR=1.03, 95% CI=0.67-1. 85, *P*=0.892).

### Stratification analysis

We further performed stratification analysis based on age, gender, and clinical stages (**Table 2**). Similarly, we did not observe any association between the rs3738067 A>G polymorphism and Wilms tumor risk in all subgroups.

## Discussion

The current knowledge of genetic predisposition to Wilms tumor is incomplete. SNPs in m^6^A-related genes are highly implicated in the risk of cancer. We hypothesized *YTHDF2* gene SNPs may also influence the risk of Wilms tumor. This pilot study provides the first indication that *YTHDF2* gene rs3738067 A>G could not impact Wilms tumor risk in Chinese children.

The final consequences of m^6^A modification on mRNA fate are executed by “reader” proteins. These proteins mainly included the YTH family (YTHDC1-2 and YTHDF1-3), HNRNPA2B1, and eIF3. YTHDF2 recognizes m^6^A mRNA within the GACU/A consensus to induce degradation of methylated transcripts 38. Cytoplasmic YTHDF1 and YTHDF3 could bind to m^6^A to initiate the translation of m^6^A-containing transcripts 39, while IGF2BP protein could enhance the stability of target mRNA 40. Growing evidence has been added to support the critical role of YTHDF2 in the regulation of cancer cell proliferation and migration. Jasmin Paris et al. 41 found that YTHDF2 is highly expressed across multiple human acute myeloid leukemia (AML) and is required for initiation and propagation in AML. YTHDF2 shortens the half-life of various m^6^A transcripts that contribute to the overall integrity of self-renewing leukemic stem cells (LSCs) function. Therefore, YTHDF2 could be treated as a unique therapeutic target for AML therapy. In hepatocellular carcinoma (HCC), YTHDF2 was found to be reversely associated with the survival of patients. Knockdown of YTHDF2 resulted in impaired stemness in liver cancer cells. Mechanistically, YTHDF2 could regulate m^6^A methylation of OCT4 mRNA and thus promote the liver cancer stem cell phenotype and HCC metastasis 42. Xie et al. 43 found that the METTL3/YTHDF2 m^6^A axis contributed to bladder cancer progression by directly degrading the mRNAs of the tumor suppressors KLF4 and SETD7. YTHDF2 was also found to be upregulated in lung cancer tissues and promotes lung cancer cell growth. Mechanistically, YTHDF2 acts as a lung cancer promoter to facilitate 6-phosphogluconate dehydrogenase (6PGD) mRNA translation through binding to the m^6^A modification site of 6PGD 44.

Contributions of the m^6^A gene to cancer are highly acknowledged, yet research on m^6^A critical gene SNPs on cancer risk is still at the primary stage. Multiple SNPs located in m^6^A critical genes have been found to impact the risk of cancer. Our previous studies also revealed the involvement of m^6^A gene SNPs in Wilms tumor 45-47. For *YTHDF2* gene SNPs and cancer risk, only one study has been conducted. In 2020, Meng et al. 48 performed the first genetic association study regarding m^6^A modification critical gene SNPs and cancer risk. They genotyped 240 SNPs within 20 m^6^A modification-related genes in samples of 2082 colorectal cancer cases and 2308 healthy controls. One SNP, rs4654320, in the *YTHDF2* gene was included in the analysis. However, all the SNPs including *YTHDF2* rs4654320 could not predispose to colorectal cancer, except for one SNP rs118049207 in the* SND1* gene. In 2012, a genome-wide association study was carried out on Wilms tumor. The authors used cases recruited through oncology clinics in North America to identify genetic variants that confer susceptibility to Wilms tumor. They selected SNPs that demonstrated an association of a significance level of *P*<5×10^-5^ for the replication phase. They failed to detect *YTHDF2* gene SNPs that were associated with Wilms tumor risk 14. Until now, no available reports have been carried out to explore the role of *YTHDF2* gene SNPs on Wilms tumor risk. Thus, here we set as a pioneer to determine the role of *YTHDF2* gene SNPs on Wilms tumor risk. The current clinical analysis indicated *YTHDF2* rs3738067 A>G could not impact Wilms tumor risk in Chinese children. We then analyzed the role of rs3738067 A>G in Wilms tumor risk using stratification analysis but still obtained negative results. Several potential reasons may help to interpret these null relationships: 1) the weak impact of SNP rs3738067 A>G; 2) the insufficient statistical power caused by moderate sample size; 3) influence of other potential pertinent factors, including modifications of environmental factors (parental exposures to pesticides, paternal occupation) 49, 50 and genetic-environmental factors.

Among the weaknesses are that the relatively small sample size of the study is underpowered to detect the weak impact of SNPs. In addition, the outcome of variant rs3738067 A>G on Wilms tumor risk was only assessed by genetic analysis. Environmental factors that greatly modified the risk of Wilms tumor remained unaccounted in the current study. Moreover, the risk variant identification here was only conducted in Chinese descendants, whether the effect of *YTHDF2* gene rs3738067 A>G can be generalized to other ethnicities needs to be confirmed. Last, the relationship was only determined in the genetic model. The relationship between *YTHDF2* and Wilms tumor from the protein level is warranted to be determined.

Taken together, our results suggested that the *YTHDF2* rs3738067 A>G polymorphism did not show a significant association with the risk of Wilms tumor in a population of Chinese children. Investigations are warranted to verify this assessment and to further evaluate the underlying role of *YTHDF2* rs3738067 A>G on the risk for Wilms tumor.

## Supplementary Material

Supplementary table.

## Figures and Tables

**Table 1:** Association between *YTHDF2* rs3738067 A>G polymorphism and Wilms tumor risk

Genotype	Cases (N=441)	Controls (N=1316)	*P* ^ a^	Crude OR (95% CI)	*P*	Adjusted OR (95% CI) ^b^	*P* ^ b^
**rs3738067 (HWE=0.860)**							
AA	253 (57.37)	731 (55.55)		1.00		1.00	
AG	158 (35.83)	498 (37.84)		0.92 (0.73-1.15)	0.457	0.91 (0.73-1.15)	0.444
GG	30 (6.80)	87 (6.61)		1.00 (0.64-1.55)	0.987	1.00 (0.64-1.54)	0.981
Additive			0.632	0.96 (0.80-1.14)	0.632	0.96 (0.80-1.14)	0.619
Dominant	188 (42.63)	585 (44.45)	0.505	0.93 (0.75-1.15)	0.505	0.93 (0.75-1.15)	0.491
Recessive	411 (93.20)	1229 (93.39)	0.889	1.03 (0.67-1.59)	0.888	1.03 (0.67-1.58)	0.892

HWE, Hardy-Weinberg equilibrium; OR, odds ratio; CI, confidence interval. ^a^ χ^2^ test for genotype distributions between Wilms tumor patients and cancer-free controls. ^b^ Adjusted for age and gender.

**Table 2:** Stratify analysis of *YTHDF2* rs3738067 A>G polymorphism with Wilms tumor risk

Variables	rs3738067 (case/control)		Crude OR	*P*	Adjusted OR ^a^	*P* ^a^
	AA	AG/GG	(95% CI)		(95% CI)	
**Age, month**						
≤18	84/272	59/228	0.84 (0.58-1.22)	0.357	0.86 (0.59-1.25)	0.431
>18	169/459	129/357	0.98 (0.75-1.28)	0.891	1.00 (0.76-1.31)	0.990
**Gender**						
Females	111/320	96/248	1.12 (0.81-1.54)	0.501	1.13 (0.82-1.55)	0.467
Males	142/411	92/337	0.79 (0.59-1.07)	0.123	0.79 (0.59-1.07)	0.129
**Clinical stages**						
I	83/731	54/585	0.81 (0.57-1.17)	0.259	0.82 (0.57-1.17)	0.274
II	70/731	49/585	0.88 (0.60-1.28)	0.491	0.88 (0.60-1.28)	0.500
III	63/731	54/585	1.07 (0.73-1.57)	0.723	1.05 (0.72-1.54)	0.809
IV	29/731	23/585	0.99 (0.57-1.73)	0.975	0.98 (0.60-1.71)	0.937
I+II	153/731	103/585	0.84 (0.64-1.11)	0.214	0.84 (0.64-1.11)	0.221
III+IV	92/731	77/585	1.05 (0.76-1.44)	0.785	1.02 (0.74-1.41)	0.901

OR, odds ratio; CI, confidence interval. ^a^ Adjusted for age and gender, omitting the corresponding variable.

## Abbreviations

LOI: loss of imprinting
LOH: loss of heterozygosity
SNP: single nucleotide polymorphism
m^6^A: N^6^-methyladenosine
MAF: minor allele frequency
UTR: untranslated region
TFBS: transcription factor binding site
HWE: Hardy-Weinberg equilibrium
OR: odds ratio
CI: confidence interval
AML: acute myeloid leukemia
LSC: leukemic stem cell
HCC: hepatocellular carcinoma
6PGD: 6-phosphogluconate dehydrogenase
