# Supplementary material for: The contribution of YTHDF2 gene rs3738067 A>G to the Wilms tumor susceptibility
Source: J Cancer. 2021 Aug 26;12(20):6165–9. doi: 10.7150/jca.62154 (PMC8425210; doi:10.7150/jca.62154)
Supplement: Supplementary file 1 — Supplementary table. [file jcav12p6165s1.pdf]

Table S1. Frequency distribution of selected variables in Wilms tumor patients and cancer-free controls from six included hospitals

Variables	Cases (N=450)		Controls (N=1317)		P [a]
	No.	%	No.	%	
Age range, month	1-168.00		0.03-156.00		0.668
Mean ± SD	32.70 ± 25.75		32.10 ± 25.36		
≤18	148	32.89	501	38.04	
>18	302	67.11	816	61.96	
Gender					0.157
Female	211	46.89	567	43.05	
Male	239	53.11	750	56.95	
Clinical stages					
I	137	30.44	/	/	
II	122	27.11	/	/	
III	119	26.44	/	/	
IV	54	12.00	/	/	
NA	18	4.00	/	/	

SD, standard deviation; NA, not available.

[a] Two-sided χ^2 test for distributions between Wilms tumor patients and cancer-free controls.

[b] T-test for age distribution between Wilms tumor patients and cancer-free controls.
